# Glucocorticoids and Preterm Hypoxic-Ischemic Brain Injury: The Good and the Bad

**DOI:** 10.1155/2012/751694

**Published:** 2012-08-16

**Authors:** Laura Bennet, Joanne O. Davidson, Miriam Koome, Alistair Jan Gunn

**Affiliations:** Fetal Physiology and Neuroscience Group, Department of Physiology, The University of Auckland, Auckland 1142, New Zealand

## Abstract

Fetuses at risk of premature delivery are now routinely exposed to maternal treatment with synthetic glucocorticoids. In randomized clinical trials, these substantially reduce acute neonatal systemic morbidity, and mortality, after premature birth and reduce intraventricular hemorrhage. However, the overall neurodevelopmental impact is surprisingly unclear; worryingly, postnatal glucocorticoids are consistently associated with impaired brain development. We review the clinical and experimental evidence on how glucocorticoids may affect the developing brain and highlight the need for systematic research.

## 1. Introduction

Since Liggins and Howie [1] showed that antenatal glucocorticoids play an important role in the prevention of respiratory distress syndrome in premature infants, antenatal glucocorticoids have been increasingly given to women at risk of premature delivery. In 1994, the National Institutes of Health Consensus Conference stated that all fetuses between 24 and 34 weeks of gestation at risk of premature delivery should be considered candidates for treatment with antenatal glucocorticoids [2]. Meta-analysis of randomized clinical trials suggests that maternal treatment with antenatal glucocorticoids substantially reduces acute neonatal systemic morbidity, and mortality, after premature birth and reduces intraventricular hemorrhage [3]. There is some evidence from individual studies that treatment may also reduce the risk of white matter injury [4, 5].

## 2. Cause for Concern?

There is limited information on the short-term and long-term effects of antenatal glucocorticoids on the brain. From animal studies there is some evidence that antenatal glucocorticoids may cause impairment of neural development [6]. These side effects include alterations of neuronal cytoskeleton and presynaptic terminals [7–9], delay in myelination [10, 11], alterations in proliferation [12], decrease in cerebral blood flow [13], alterations in neuronal activity [14], and brain growth retardation [15].

In humans, antenatal glucocorticoids were associated with an increased risk of disorders in aggressive destructive behaviour, hyperactivity, and distractibility [16]. However, results are not consistent in different studies. MacArthur and colleagues [17, 18] found no effect of betamethasone treatment on cognitive development of 4-year-old children and cognitive development and school progress of 6-year-old children. After a 10- to 12-year followup, children who were treated with antenatal glucocorticoids did not differ from controls in growth, lung function, development of sexual characteristics, sleep quality, incidence of physical anomalies, and neurological status [19]. Murphy found no brain growth retardation in babies delivered preterm who died [20].

In contrast, there is now considerable experimental and clinical evidence that postnatal administration to preterm infants is consistently associated with impaired brain development [21]. In rodents glucocorticoids are associated with reduced proliferation and a decrease in total cell numbers, leading to impaired brain growth and development [5, 7–9, 12, 15, 22–28]. Indeed, in rodents exposure to glucocorticoids before or immediately after birth may be associated with marked upregulation of neuronal and progenitor cell apoptosis, particularly in the hippocampus where they deplete the pool of neural progenitor cells and reduce hippocampal growth [29–31]. In turn, there is increasing evidence that exposure of neonates to exogenous glucocorticoids impairs memory and increases the risk of CP [32].

Pragmatically, the effects of prenatal and postnatal exposure are of course unlikely to be identical. The duration of maternal treatment is much shorter (typically 2 to 4 doses over 24 h versus 3 to 42 days of daily treatment [33]) and given the role of the placenta, it is likely that the profile of fetal plasma concentrations would be lower than after maternal administration compared with postnatal treatment. Nevertheless, these data strongly suggest that it is vital to understand how glucocorticoids affect the developing brain [22].

## 3. Cerebral Palsy after Preterm Birth

Cerebral palsy (CP) associated with premature birth now accounts for over a third of total cases [34]. The costs to society are huge. CP has one of the very highest indices of burden of disease from loss of potential productive members of society and direct burdens on the individual, family, and social institutions that last the entire life [35]. In the USA, 13% of all births are preterm, costing over $26.2 billion in 2005 alone [36]. There has been little or no reduction in the incidence of CP of perinatal origin, and the risk may even have increased in some Western countries [36], despite 35 years of advances in perinatal care. Most of these costs are related to long-term neurodevelopmental disability in surviving infants, rather than acute care after birth [37–39]. Although there has been a reduction in the most severe form of cystic white matter injury over time [40], even subtle injury is associated with impaired brain development and disability [38, 41]. It is striking that risk of injury and disability are increased sevenfold even in “late preterm” infants of 34 to 36 weeks gestation [42–45]. Thus, even small changes in the risk of disability would have substantial real-world impact.

## 4. What Causes Injury after Preterm Birth?

Although the etiology of neurodevelopmental disability after preterm birth is undoubtedly multifactorial, there is considerable evidence that exposure to perinatal hypoxia-ischemia (HI) is a major trigger [46]. In turn, this may be augmented or modulated by prior exposure to hypoxia, infection/inflammation, seizures, and placental damage [47–52]. In addition, babies are exposed to many clinical interventions. Notably, unborn children are now routinely exposed to maternal treatment with potent synthetic glucocorticoids. Although steroid treatment is one of the most cost effective, live-saving treatments in fetal medicine, it is not without potential risks for some fetuses.

## 5. Betamethasone versus Dexamethasone

It has been much debated whether there is any material difference in neonatal outcomes between two major synthetic glucocorticoids used for antenatal treatment, despite these compounds differing only in the orientation of one methyl group. For example, it has been suggested that betamethasone, but not dexamethasone can reduce the risk of periventricular leukomalacia in very premature infants [4]. Further, betamethasone has been associated with better survival and reduced risk of adverse outcomes [53], and with better neurodevelopment and fewer hearing problems [54]. In contrast, others found no differences [55, 56], and one study suggested that dexamethasone reduced the risk for intraventricular hemorrhage more than betamethasone [56]. Conversely, betamethasone may have more pronounced effects to suppress fetal movements and heart rate variation [46, 47]. Overall, there is a striking lack of information about the effects of these steroids on neural outcomes or fetal responses to hypoxia, and few formal comparisons have been performed.

## 6. The Effects of Glucocorticoids on Postnatal Hypoxic-Ischemic Brain Injury

The effect of glucocorticoids during and after HI in rodents appears to be rather variable. Clinically, glucocorticoids are used therapeutically to reduce brain edema, but clinical trials of glucocorticoids in ischemic stroke, intracerebral haemorrhage, aneurysmal subarachnoid haemorrhage, and traumatic brain injury have not shown any clear therapeutic effect [57]. Experimental studies of ischemia in adult rats or *in vitro* have generally suggested increased injury when glucocorticoids are given before the insult (range 1 to 48 h beforehand) [58–65]. Immediate pretreatment was reported to have no effect, whereas chronic postinsult treatment reduced injury in the caudate nucleus, but had no effect on other brain areas [60].

In contrast, in newborn rats at postnatal day 7 (P7), when brain maturation is equivalent to the mildly preterm infant [66, 67], preinsult treatment (4 to 48 h beforehand) significantly reduced injury [68–74]. In contrast, glucocorticoids were not protective if given just before (0 to 3 h) or after HI [68, 75], and post-HI treatment increased mortality [75]. Antenatal treatment (on embryonic days 17, 18, and 19) has also been associated with reduced survival during hypoxia on the day after birth [76].

Injury may be mediated by reduced glucose uptake and energy utilization, accelerated ATP loss, and excessive Ca^2+^ mobilization during ischemia, and augmented excitotoxic and inflammatory responses post-ischemia [59, 61, 62, 77–80]. Conversely, neuroprotection has been related to stabilizing ATP production, with maintenance of glycolytic flux [70, 81, 82]. There may be maturation dependent responses to glucocorticoids, dependent in part on cerebral metabolism [83–85]. In support of this, preischemic hyperglycemia reduced injury in P7 rats due to energy preservation [86–88].

However, this may well be a species dependent response as hyperglycemia increased injury in newborn piglets, despite energy preservation [89]. Postischemic hyperglycemia was not protective in either species [90, 91]. In adult animals glucose can increase, reduce or have no impact on injury [58, 59, 92–99], and clinically it remains highly controversial whether controlling glucose affects outcomes after ischemia [100]. Nevertheless, maternal diabetes is associated with adverse neurodevelopmental outcomes in children, and this is strongly correlated with maternal hyperglycemia [101]. Further, hyperglycemia in preterm infants is associated with increased morbidity and mortality [102].

## 7. How Do Glucocorticoids Affect the Fetus?

**Figure 1:**
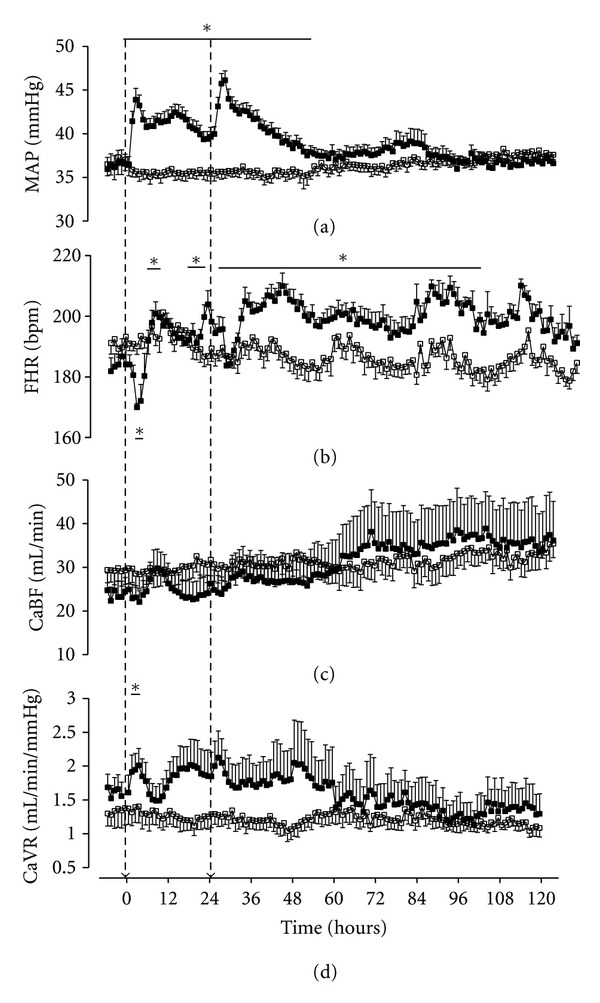
Time sequence of changes in mean arterial pressure (MAP), fetal heart rate (FHR), carotid blood flow (CaBF), and carotid vascular resistance (CaVR) in 0.7 gestation fetuses after two maternal intramuscular injections of either saline (open squares, *n* = 7) or dexamethasone (filled squares, *n* = 8). Dexamethasone induced a significant increase in MAP after each injection, and pressure was elevated for 50 hours. FHR fell acutely after each injection, and there was a sustained period of tachycardia after the second injection. There was a tendency for CaBF to fall initially, and this was associated with a significant rise in resistance. However, overall there were no marked differences in cerebral perfusion and resistance. Data are shown from six hours before the first injection to 120 hours afterwards. The dashed arrows show the timing of injections. Data are one hour averages, mean (SEM), *P < 0.05.

**Figure 2:**
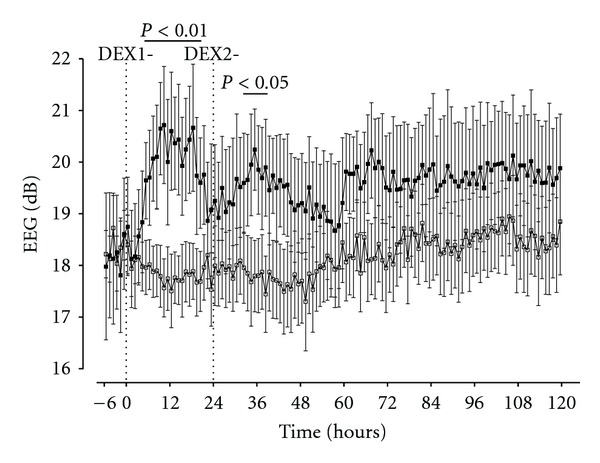
Time course changes in EEG power (dB, decibels) before and after two injections of dexamethasone (filled squares) or saline (open squares) 24 hours apart. The injections for each group are marked by dotted lines (DEX-1 and DEX-2). A significant increase in EEG power was seen after both DEX-1 and DEX-2, but the effects of DEX were most significant after the first injection. Peak amplitude changes were around 12 hours, and the increase in amplitude was mediated by an overall increase in normal sleep architecture waveforms and the appearance of seizures (see Figure 3). Data are one hour means ± SEM from six hours before until 120 hours after DEX-1.

**Figure 3:**
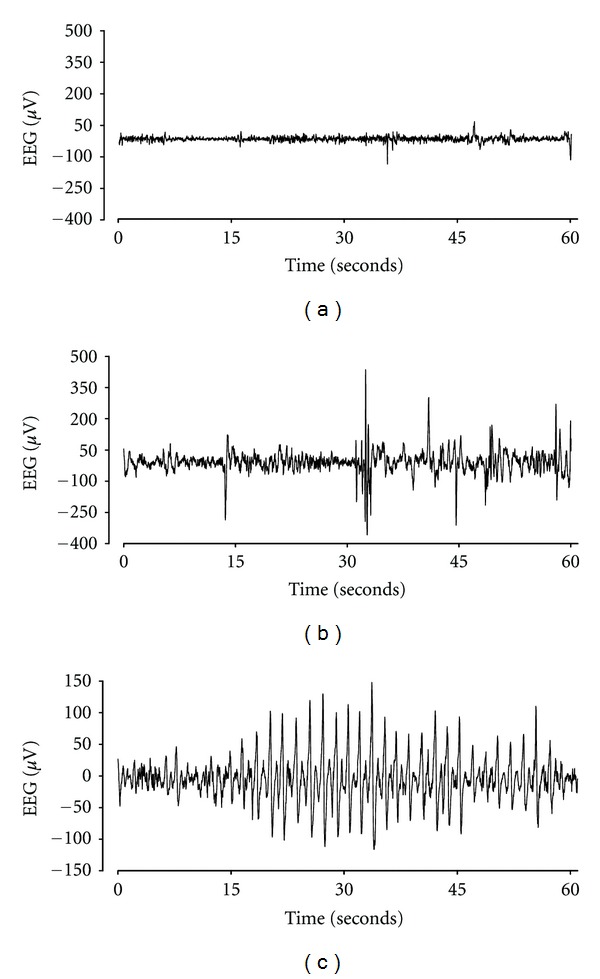
Example of sleep state architecture in a 0.7 gestation preterm fetal lamb before exposure to maternal dexamethasone (panel (a)) and after dexamethasone (panel (b)). The normal sleep patterns of fetal sheep at this age is composed of mixed EEG amplitudes and frequencies, with mean amplitude typically around 60–70 *μ*V. Transient waveforms (fast, sharp, and slow waves between 70–400 ms) are seen as part of this normal EEG patterns and their amplitude is typically between 70–150 *μ*V. After dexamethasone exposure, there is a significant change in sleep architecture characterised by a significant increase in EEG amplitude (with background amplitudes increased to above 100 *μ*V and spikes ranging between 200–600 *μ*V), and a general reduction in high-frequency activity. Seizures and seizure-like activity is also observed and an example of this is shown in panel (c). While many of these seizures were high amplitude, it is notable that many were relatively low amplitude. All figures are raw data from one animal.

Glucocorticoids are well documented to acutely affect fetal behaviour and cardiovascular function [103–106]. For example, a single clinical course of maternal dexamethasone in sheep at 0.7 of gestation, when brain development is broadly consistent with the 28 to 32 week human fetus [107], was associated with an acute, transient increase in mean arterial blood pressure with a rise in carotid and femoral vascular resistance, a fall in femoral arterial blood flow, and a brief fall in fetal heart rate followed by tachycardia (Figure 1) [106]. There was a persistent loss of the circadian pattern of fetal heart rate variability 3 days after maternal dexamethasone. Similarly, in pregnant sheep near-term, when fetal brain development is broadly comparable to the term human [107, 108], a clinical regime of betamethasone was associated with reduced fetal cerebral perfusion and increased cerebral metabolic activity [109]. In preterm fetal sheep, a clinical dose of maternal dexamethasone reduced the number and duration of interburst intervals [110], with a commensurate increase in the continuity of the fetal EEG, suggesting that glucocorticoid exposure increased the functional maturation of the brain (Figures 2 and 3). Other effects include the intriguing finding in near-term fetal sheep that exposure to dexamethasone increased Na-K-ATPase activity and tight junction expression [111, 112]. This may be of significance, since in neonatal rats, treatment with dexamethasone before HI was associated with both reduced blood brain barrier leakage and reduced HI brain injury at P7 and P14, but not P21 [103].

There are no data on whether glucocorticoid exposure may affect the preterm fetal ability to adapt to hypoxia. However, in near-term fetal sheep maternal dexamethasone (2 maternal injections 24 h apart) before moderate isocapnic hypoxia, which does not cause overt neural injury, was associated with impaired cardiovascular responses, with significant bradycardia and increased acidosis during hypoxia 8 hours after the second injection, whereas there was no effect 3 days after the second injection [113, 114]. Despite suppression of hypothalamic-pituitary axis function [115], low doses of dexamethasone augmented the glycemic response to hypoxia in near-term sheep fetuses [116].

## 8. The Effects of Glucocorticoids on Brain Activity in Preterm Fetal Sheep: A Potential Role for Conditioning?

We recently demonstrated that a clinical course of maternal dexamethasone was associated with significant dysregulation of brain activity in healthy preterm fetal sheep, including a loss of the normal gating of EEG waveform amplitude, and induction of seizures (Figures 2 and 3) [110]. In this paradigm, fetal blood glucose transiently increased, peaking ~4–6 h after maternal DEX treatment [106, 110]. There was no cerebral injury after 5 d of recovery. Seizures in early life in the rat [117], and other settings [118], can induce neuroprotection against later injury through preconditioning mechanisms, for example, by inducing protective gene changes. Thus, it is plausible that this activity could be beneficial given the serious neural risks of preterm birth [36].

The caveat to this reassuring hypothesis is that after exposure to preconditioning insults, such as seizures, it typically takes several days before protection is established [119–122]. If there are only a few hours between the stimulus and the HI insult, then sensitization occurs [119–122]. Thus, injury may occur with an otherwise noninjurious insult. In this setting both hyperglycemia and greater transient activity after glucocorticoid exposure could make the brain more vulnerable to HI. Epileptiform transients are associated with greater injury after HI in the preterm fetal sheep [123], likely by increasing the metabolic stress of injured cells making them vulnerable to further hypoxia [124]. Hyperglycemia increases glucose utilisation in the fetus [125], increasing neural metabolism and the metabolic demand on sick cells [126, 127]. Again, although these data are not definitive, they suggest potential for risk.

## 9. Glucocorticoids and HI in the Fetus

Rather remarkably, given the established clinical use of glucocorticoids for threatened premature delivery, we have been able to identify only one study of their effects on HI injury in the fetus. In near-term fetal sheep, Ellit and colleagues reported that chronic pretreatment (48 h before ischemia induced by carotid artery occlusion) had no effect on subsequent ischemic brain injury [128]. In contrast, in cultured embryonic rat basal forebrain cells, exposure to dexamethasone before oxygen-glucose deprivation was associated with increased injury [77]; this damage could be negated by glutamate receptor blockade. Potentially these contrasting results between protection in rats, no effect in sheep, and increased injury *in vitro*, might be related to species or to the specific model of brain injury, or to lower plasma levels after maternal treatment compared to direct neonatal injection. It is also critical to appreciate that the dose response for the glucocorticoid effects on neuronal cell viability and survival is both narrow and U-shaped [129].

## 10. Conclusions

Given the frighteningly limited information on whether glucocorticoids protect or sensitise to HI brain injury in preterm fetuses [130] it is of considerable concern that glucocorticoids have been suggested to be potential perinatal neuroprotection agents [84, 85]. The recent finding discussed above that a clinical course of maternal dexamethasone exposure triggers major changes in brain activity, including epileptiform events now raises the possibility that maternal glucocorticoids may well be both protective and damaging, depending on the precise timing before the preterm fetus and infant are exposed to an HI insult. These concepts suggest that as with so many aspects of neonatology timing is everything! [131] In turn, variations in timing of exposure may well explain the rather variable effects of maternal treatment in human clinical trials suggested above. Further systematic clinical and basic science studies are essential to tease out these factors.
